# Prevalence and Progression of Gingivitis in Domestic Cats in Subtropical Mexico

**DOI:** 10.1155/2024/6611814

**Published:** 2024-03-21

**Authors:** Ana G. Villegas-Ferré, Víctor Martínez-Aguilar, Samantha Valencia-Arjona, Eduardo Gutiérrez-Blanco, Matilde Jiménez-Coello, José C. Segura-Correa, Antonio Ortega-Pacheco

**Affiliations:** ^1^Autonomous University of Yucatan, Faculty of Veterinary Medicine and Animal Science, Km 15.5 Carr, Mérida-Xmatkuil, Merida Yucatán, Mexico; ^2^Autonomous University of Yucatan, Faculty of Odontology, C.61A x Av. Itzaes, Mérida Yucatán, Mexico

## Abstract

With the objective to characterize the gingival index (GI) and its progression, 218 domestic cats in a subtropical region of Mexico were studied. All teeth of each cat were examined with a periodontal probe to determine the GI; in addition, the absence of teeth was recorded. Six months later, the teeth of the 38 cats were again examined to assess any progression of the GI and loss of teeth. From the 218 cats, 33.0% of them develop some degree of gingival inflammation; from those, 61.5% were classified as GI 1. Age, sex, and neutered status were associated with tooth affections. Missed teeth were observed in 35% of the cats, particularly for molars 109 and 209 in both sexes. After six months, the number of teeth with GI 1 decreased to 20%. The gingival problems in cats have not been well studied, particularly at the speed they progress and how this can affect the loss of teeth; under the conditions of this study, a high frequency of gingival inflammation even at early age was demonstrated, with a rapid tooth loss. Although young males were more prone to develop gingivitis, females tend to loss more teeth. Non-neutered cats tended to develop more dental affections.

## 1. Introduction

There is limited information on the epidemiology of gingivitis in domestic cats even though this is a common condition of the oral cavity in this species [1]. Gingivitis is the inflammation of the gingival tissue without destruction of the materials that involve the dental support. It is caused by a plaque attached to the dental surfaces, which is considered a biofilm made up almost entirely of oral bacteria [2]. Gingivitis in cats can reach a very high prevalence, being able to affect up to 70% of young cats (1.8–2.3 years of age) and up to 85% when they are older than 6 years [3, 4]. A study on gingivitis and periodontitis in cats reports that of 109 animals evaluated, 13.0% had severe periodontitis, and all of them had some grade of periodontal inflammation [5]. In a more recent study with free roaming cats (“community cats”), gingivitis was detected in 13.3% of them with a prevalence of 53.3% of incisive dental losses [6]. There are several risk factors associated with the development of these conditions. Age seems to be the main factor because older cats are more susceptible to the development of oral pathologies; diet also plays a role in their development, cats on a wet or soft food diet seem more susceptible to gingivitis and periodontitis than cats on a hard food diet [7]. Bacteria also play an important role in the onset and subsequent development of gingivitis and periodontitis, participating in the formation of the periodontal pocket, connective tissue destruction, and alveolar bone resorption by means of an immunopathogenic mechanism. On the other hand, control of plaque formation and gingivitis can be achieved by using gel dental products [8] and toothbrushing [9] and in chronic cases, by tooth extraction [10]. However, in many cases, tooth care is not feasible in cats because the patients are not compliant or because this is not a common practice in cat owners, particularly in developing countries.

To our knowledge, no previous studies describing the progression of gingival indices in cats or their effect on dental integrity have been reported, particularly in tropical or subtropical regions. Therefore, the objective of this study was to determine the prevalence and progression of gingival indices in domestic cats in a subtropical region of Mexico.

## 2. Materials and Methods

### 2.1. Study Area

The study was carried out in the cities of Merida (20°58′N and 89°37′W) and Progreso (21°16′ N and 89°39 W), located in the Yucatan Peninsula, Mexico, with a subhumid tropical climate and rains during the months of May and June, October, and November [11]. The study was carried out with patients referred to the Faculty of Veterinary Medicine and Animal Science, from private clinics, a feline shelter, and with cats from different spaying campaigns.

### 2.2. Sample Size

Considering the nature of studied cats (owned but free roaming and with no dental care), the sample size was calculated considering an expected prevalence of 13% of cats suffering from moderate to severe gingivitis [5], assuming an infinite population, a 95% confidence level and 5% precision, which gave at least 174 animals.

### 2.3. Animals and Inclusion Criteria

Cats living in Merida and Progreso, of both genders, with a minimum age of 1 year (estimated based on the patient's history and/or their dentition), of any breed and size, and without history of dental prophylaxis were included. A brief questionnaire was applied to the owners to obtain information on diet, health, reproductive status, and lifestyle. The prevalence of the gingival index was estimated from the total sample size of animals. After six months, 38 cats were again examined and followed to evaluate the progression of gingivitis and dental loses; before the examination, an informed consent was signed by the owner. The protocol of this study was approved by the Bioethics Committee of the Biological and Agricultural Sciences Campus, “Universidad Autónoma de Yucatán” (CB-CCBA-M-2019-012).

### 2.4. Gingival Examination

Prior to sedation, a clinical examination was performed on each cat to ensure that they were in good health. A premedication with 5 *μ*g of dexmedetomidine i.m. (Krunamina® Vet, PiSA, Mexico City, MX) followed by a mixture of tiletamine hydrochloride and zolazepam hydrochloride (Zoletil, Virbac, Guadalajara, MX) at a dose of 4 mg/kg i.m. was used. Once the cat adopted lateral recumbence, the inspection of the oral cavity was performed. After examination, patients were awaked with an i.m. injection of atipamezole at a dose of 25 *μ*g (Antisedan, Zoetis, Mexico City).

### 2.5. Gingival Index (GI)

The GI was measured based on the criteria previously described [5]. In brief, the four points of the gingiva (distal, mesial, buccal, and lingual) for each tooth were evaluated based on the clinical characteristics of the different grades of gingivitis. A probing of the gingival margins and their depth was carried out using a William's periodontal probe (Medesysrl, Maniago, Italy) and recorded on periodontogram designed for this purpose. The GI used is described in Table 1. Because only gingival lesions were explored and not periodontal disease, X-rays were not used.

The scores of the four areas of the tooth were divided by four to have the GI of each tooth. The missing of mandibular and maxillary teeth was also noted.

### 2.6. Follow-Up Examination

After 6 months, evaluation of the oral cavity was carried out in 38 cats to determine if the GI have progressed, decreased, or kept without change; in addition, the loss of teeth was also registered. As in the first dental examination, patients were sedated and the gingival index was assessed. Because some of the cats died, other disappeared in the previously study period (6 months) and several owners were reluctant to allow a second examination, only 38 cats were followed up.

### 2.7. Statistical Analysis

The GI was calculated as the average of the 218 cats according to the degree of involvement. The GI was also estimated by age group (1-2 and ≥3-4 years old), sex (male and female), and reproductive status (neutered or non-neutered). In addition, odds ratio and confidence intervals by risk factor were estimated and Chi-square tests were carried out to detect statistical differences (*p* < 0.05).

## 3. Results

The oral cavity of 218 cats was evaluated to assess the GI. Sixty-five percent of the cats (*n* = 142) were female and 35% (*n* = 76) were male, of which 24.8% were neutered (*n* = 54) and 75.2% were non-neutered (*n* = 164). In 95.9% of the cases, cats were fed a commercial dry food and 87% were allowed to freely roam outside the house.

### 3.1. Gingival Index

**Figure 1:**
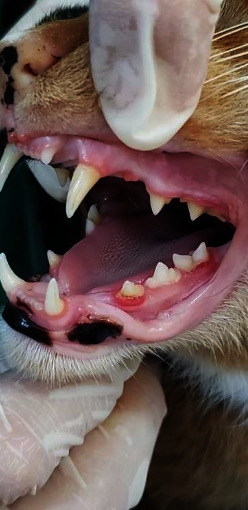
An erythematous area is seen along the gingiva in the left maxillary region; the mandibular premolar 307 and molar 309 show obvious inflammation at their base with the presence of calculi, particularly in premolar 307.

**Figure 2:**
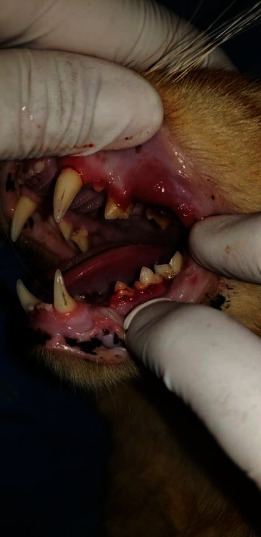
A severe gingivitis is noted along the maxillary and mandibular regions with bleeding on probing; premolars 207 and 208 shown gingival enlargement over a resorptive lesion.

Thirty-three percent of the cats showed a GI0 grade (they did not have gingivitis); 61.5% showed a IG 1 grade; 5% showed IG 2 grade, and 0.5% had GI 3 grade.  Table 2 shows the frequency of the GI and the age group. Cats with ages from 1 to 2 years showed a higher frequency of GI 0 and GI 1. In most of the cats, GI 1 (61.5%) was the predominant affection in 1-2 years old cats. Thirty-three percent of the cats had no lesions (GI 0), and GI 2 and GI 3 cases were observed in 5.0% and 0.5% of evaluated cats, respectively. Young cats (1-2 years old) were 22.1 more likely to develop GI 1 than older cats (*p* < 0.000); males, 2.5 more likely than females (*p* < 0.002); and non-neutered cats, 2.6 times higher to develop GI 1 than neutered ones (*p* < 0.003; Table 3). Examples of GI 2 and GI 3 are given in Figures 1 and 2, respectively.

### 3.2. Missing Teeth

Thirty-five percent of the cats examined had at least one-loss teeth. The most frequent dental losses were observed in the maxillary molars, 109 and 209 for females and males, respectively (Table 4). Regarding the premolars, the piece with the greatest absence corresponds to 106. The maxillary incisors with the highest tooth loss were the centrals 101 and 201. With respect to the maxillary teeth, the incisors 401, 301, and 302 were the pieces where more losses were observed.

### 3.3. Follow-Up Examination

#### 3.3.1. Gingival Index

**Figure 3:**
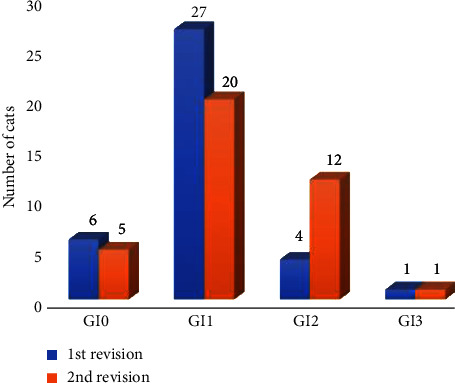
Comparison of gingival indices in 38 cats over a period of 6 months. GI0 = normal gingiva; GI 1 = mild inflammation; GI 2 = moderate inflammation; GI 3 = severe inflammation.

In the follow-up study (*n* = 38 cats), changes in the GI grade were observed from the first to the second evaluation (6 months later). A decrease of GI 0 and GI 1 grades were observed, whereas GI 2 increased from 4 to 12 cases in the second evaluation (Figure 3).

#### 3.3.2. Teeth Loss

**Figure 4:**
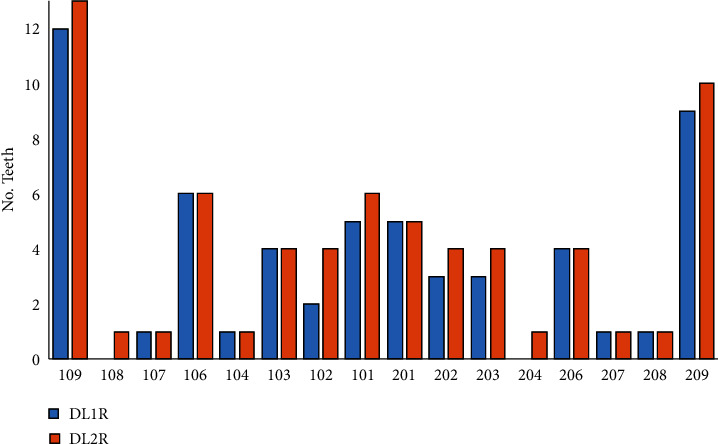
Maxillary dental losses in 38 cats in a 6 -month period. DL1R = dental losses in first revision. DLR2 = dental losses in second revision.

**Figure 5:**
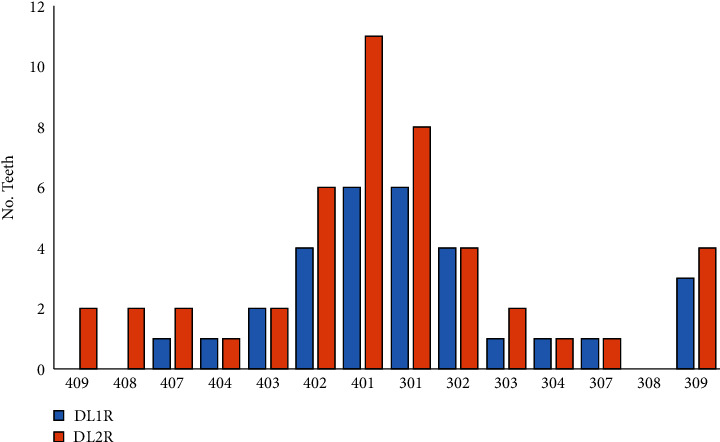
Mandibular dental losses in 38 cats in a period of 6 months. DL1R = dental losses in first revision. DLR2 = dental losses in second revision.

In the follow-up study, the loss of several teeth was observed. The maxillary teeth with the highest losses were molars 109 and 209, which increased 2% of the losses in both pieces. Premolars 108 and 204 had a 3% increase in teeth loss (Figure 4). Mandibular molars 409 and 309 and premolars 407 and 408 showed a 5% increase of losses. The incisors pieces with the most dental losses were 402, 401, and 301 with 5% (Figure 5).

## 4. Discussion

Gingivitis occurs after 2-3 weeks if the plaque biofilm is not eliminated, allowing the entry of bacteria and their products to the gingiva. This eventually may cause vasodilation and even bleeding on probing, and if it is not attended, it develops into a state of chronic inflammation and formation of periodontal pockets [1]. A review of the available literature found that there are no systemic studies on feline gingivitis in Mexico. Literature describes that gingivitis in cats has a high prevalence, being able to affect 70% of young cats of 1.8–2.3 years of age [12] and up to 85% in 6 or more years of age cats [13]. In a study in a colony of 109 cats, a high prevalence (13%) of gingival inflammation was found [5]. The present study confirms the high prevalence of gingivitis in cats studied under subtropical conditions, with a degree of gingival inflammation that in most cases was mild (GI 1). When gingival pockets are present, the health of the mucosal surface could be easily affected. However, although gingivitis occurs spontaneously in cats [12], some factors may predispose it. Gingival inflammation can be accelerated by factors that enhance plaque accumulation [14]. It includes crowding [15], roughened teeth due to fracture, enamel hypoplasia, or aggressive scaling [16]. In addition, diseases (i.e., feline immunodeficiency virus) that alter the host inflammatory response may predispose the patient to the development of gingivitis [17]. In the present study, since diets were similar in most of the evaluated cats, it was not considered as a risk factor. However, age seems to be a predisposing factor for the presentation of gingivitis. In addition, as shown here, young animals between 1 and 2 years of age develop gingival inflammation. This inflammation at a young age may be due to a syndrome called juvenile periodontal disease, which is the inflammation of the gingiva occurring shortly after the permanent dental eruption [1, 2]. If the initial gingivitis is not relieved, the inflammation can progress and be more severe particularly if a bacterial plaque develops. Results from the present study indicates that severe cases of gingival inflammation are less frequent, but the limited number of cats >5 years old studied here may not allowed for such postulation. Unlike other studies [5, 18, 19], the sex of the cats seems to be associated with a predisposition to gingivitis. Males had the most predisposition although there is not a precise explanation. However, free roaming and ethological habits of cats (such as dominance, hunting, competition, and variety of food) may predispose to teeth lesions and formation of bacterial plaque. However, this cannot be established with the data collected in the present study.

Neutered cats were less likely to develop gingivitis than non-neutered cats. However, although sterilization can have a favorable effect on the health of cats [20], the information collected in the present study did not provide enough elements to explain the biological reason for this association.

Gingivitis in cats may also be associated with viral infections causing mucosal inflammation, including feline leukemia virus, feline immunodeficiency virus, and feline calicivirus [21]. However, in the present study, this was not evaluated but should always be considered when dealing with cats suffering of gingivitis.

The results of this study showed that 35% of the teeth were loss, which agrees with the range of 20%–56% reported by other authors [5, 18, 22]. However, the etiology of such losses is not well known. It was found that the most frequent missing teeth at the maxillary site were molars 109 and 209 in both males and females; in this case, both teeth can be vestigial and are not always present in all cats. Those teeth can also show morphological variations as single rooted that predispose them to be easily lost [23]. Tooth resorption formerly known as feline odontoclastic resorptive lesion (FORL) is a common condition seen in cats, with reported prevalence up to 37.5% [24]. This is a progressive loss of tooth substance associated with damage to the cementoblast layer, periodontal ligament, or neurovascular bundle. As time goes, all areas of the affected tooth, from root to crown, may become involve. In one study, the most commonly affected teeth with tooth resorption were the mandibular third premolars (307 and 407) [25], and in another study, the premolar 407 showed a greater tendency to develop tooth resorption [26]. In the present study, only the presence or absence of teeth was evaluated, so tooth resorption could not be diagnosed. The missing of a high number of mandibular and maxillary incisors, therefore, is not clear. Dental resorption in cats has been reported in the canines and incisors of 82.14% and 75.0% of the maxillary and mandibular regions, respectively. This lesion is characterized by having vascularized granulation tissue bordering the cementum or, in advanced cases, to dentin; therefore, these teeth are likely to be more susceptible to periodontitis [12]. In the present study, the incisors were, together with the molars, the teeth with the highest periodontal index because incisors are small teeth located between the canines and are used for prehension. The increased prevalence of resorption in the incisors might be associated to several aspects such as altered feeding practices, vaccination, and neutering programs [27]. Since loss of incisor teeth was more commonly found in females, it may also be associated to the temporarily loosen of ligaments and bones during pregnancy or more predisposition to develop gingivitis reported in humans [28]. Dental integrity during clinical evaluation of cats suffering of gingivitis and periodontal disease should include intraoral X-ray to monitor the degree and progression of lesions to decide the need of a special therapy, dental extraction, or the outcome of the teeth involved.

A reduction in the GI 1 was observed from the first to the second revision performed 6 months later, but this was probably due to the loss of the teeth affected with gingivitis. The more affected teeth got worse even when there were dental losses; this is because the predisposing factors were not corrected and there was a progress of the disease. There are no published reports of follow-up studies on gingivitis in cats. Due to the normal course and progression of gingival disease observed in the cats studied, dental losses, particularly of the mandibular incisors, remained constant. However, the occurrence of dental resorptions is also considered. More detailed investigations including radiographic studies should clarify the origin and morphological changes in the loosed teeth in such a shorter period of time, as previously reported [29].

## 5. Conclusions

The studied cats under subtropical conditions developed a rapid gingivitis even at young age, particularly in males; besides, dental losses are very common in the maxillary incisors, probably associated with the gingivitis. Further studies must be carried out to find the possible causes of gingival damage using a more robust sample size, including radiographical evaluation.

## Figures and Tables

**Table 1:** Description of the gingival index (GI) used to evaluate cats.

Gingival index	Description
GI 0	When the gingiva was normal (absence of inflammation, no bleeding)
GI 1	When there was mild inflammation, including slight erythema, minimal superficial alteration, and no bleeding on probing
GI 2	When there was moderate inflammation, including erythema, and delate bleeding on probing
GI 3	When there was a severe erythema and swelling, tendency to spontaneous bleeding, possible ulceration, and spontaneous bleeding on probing

**Table 2:** Frequency of the gingival indices and the age group of the 218 cats.

Age interval (years)	Total *n*	GI 0 *n* (%)	GI 1 *n* (%)	GI 2 *n* (%)	GI 3 *n* (%)
1-2	161	58 (36.1%)	97 (60.2%)	6 (3.7%)	0 (0%)
3-4	48	12 (25.0%)	32 (66.7%)	4 (8.3%)	0 (0%)
>5	9	2 (22.2%)	5 (55.6%)	1 (1.1%)	1 (1.1%)
Total	218	72 (33.0%)	134 (61.5%)	11 (5.0%)	1 (0.5%)

*n* = number of cats; GI = gingival index.

**Table 3:** Frequency of GI1 by age, sex, and reproductive status from 134 domestic cats.

Variable	Total	Positives	%	OR	95% CI	*P* value
*Age group*						
(i) 1-2	161	126	78.3	22.1	9.51–51.11	0.000
(ii) ≥3	57	8	14.0	1	—	

*Sex*						
(i) Male	76	57	75.0	2.5	1.36–4.70	0.002
(ii) Female	142	77	54.2	1	—	

*Reproductive status*						
(i) Neutered	54	24	44.4	1	—	0.003
(ii) Non-neutered	164	110	68.3	2.6	1.35–4.79	

OR = odd ratio; CI = confidence interval.

**Table 4:** Comparison of dental losses between males and female cats according to their dental formula.

		Right maxillary dental formula								Left maxillary dental formula							

		109	108	107	106	104	103	102	101	201	202	203	204	206	207	208	209

Number of cats (*n*) (%)		29 (13.3)	1 (0.5)	3 (1.4)	18 (8.4)	1 (0.5)	7 (3.2)	10 (4.6)	17 (7.8)	20 (9.2)	10 (4.6)	8 (3.7)	2 (0.9)	9 (4.1)	4 (1.8)	3 (1.4)	24 (11)

Sex (*n*) (%)	F	16 (55)	1 (100)	2 (67)	12 (67)	1 (100)	4 (57)	5 (50)	8 (47)	13 (65)	6 (60)	4 (50)	1 (50)	8 (88)	3 (75)	3 (100)	15 (63)
	M	13 (45)	0 (0)	1 (33)	6 (33)	0 (0)	3(43)	5 (50)	9 (53)	7 (35)	4 (40)	4 (50)	1 (50)	1 (12)	1 (25)	0 (0)	9 (37)

Number of cats (*n*) (%)			3 (1.4)	0 (0)	2 (0.9)	1 (0.5)	9 (4.1)	14 (6.4)	28 (12.8)	30 (17.4)	27 (12.4)	8 (3.7)	2 (0.9)	3 (1.4)	0 (0)	5 (2.3)	

Sex (*n*) (%)	F		3 (100)	0 (0)	2 (100)	1 (100)	6 (67)	8 (57)	20 (71)	18 (60)	18 (67)	6 (75)	1 (50)	2 (67)	0 (0)	4 (80)	
	M		0 (0)	0 (0)	0 (0)	0 (0)	3 (33)	6 (43)	8 (29)	12 (40)	9 (33)	2 (25)	1 (50)	1 (33)	0 (0)	1 (20)	

			409	408	407	404	403	402	401	301	302	303	304	307	308	309	

			Right mandibular dental formula							Left mandibular dental formula							

## Data Availability

The datasets used and/or analyzed during the current study are available from the corresponding author upon request.
